# Books Received

**Published:** 1869-02-15

**Authors:** 


### BOOKS RECEIVED.

OPTHALMIC SURGERY AND TREATMENT; WITH ADVICE ON THE USE AND ABUSE OF SPECTACLES. By JOHN PHILLIPS, Optician and Occulist. Chicago: *The Western News Company*; *W. B. Keen & Co.* ,1869. Pp. 510.

The first portion of the book is a complete thesis on "the use and abuse of spectacles," and is eminently practical in its character, and we think well calculated to impart knowledge on a very important and much-neglected subject. It will bear careful perusal. The various inflammatory diseases of the eye naturally claim a large space. As a whole this work is well written, and especially is this the case in the chapters on scrofulous opthalmia, and conjunctivitis and its sequel, "granulated eyelid," the most frequent and by far the most obstinate "of the curable diseases to which the eye is subject." The various formula in this part of the work will be found valuable.

The chapter on diseases of the lachrymal apparatus (25 pages) is well written, and exhausts the subject. Due credit is given to Mr. Bowman for the introduction of his simple and successful operation of slitting up the puncta and canaliculi, and adopting a series of large probes in the treatment of obstructions and strictures of these passages.

The division of the work on opthalmic surgery is, of course, about the same as in other recent works on the same subject, but as no branch of surgery is more progressive than this, and improvements, both in instruments and the *modus operandi*, are constantly being made, it is something to say that this work is, in some respects, more comprehensive, and in advance of its predecessors.

The new work of Dr. Bader, just published in London, contributes a fair quota of valuable and reliable matter.

We notice that Dr. Trumbull's practice (introduced in 1837, and since fallen into disuse) of treating various maladies of the eye by exposing the organ to medicated vapors, is here strongly advocated, and we remember that Dr. Williams, of Boston, in his recent work, takes similar ground, especially in the treatment of asthenopia.

